# Supplementary material for: Pro-inflammatory hepatic macrophages generate ROS through NADPH oxidase 2 via endocytosis of monomeric TLR4–MD2 complex
Source: Nat Commun. 2017 Dec 21;8:2247. doi: 10.1038/s41467-017-02325-2 (PMC5740170; doi:10.1038/s41467-017-02325-2)
Supplement: Supplementary file 1 — Supplementary Information [file 41467_2017_2325_MOESM1_ESM.pdf]

Supplementary Information

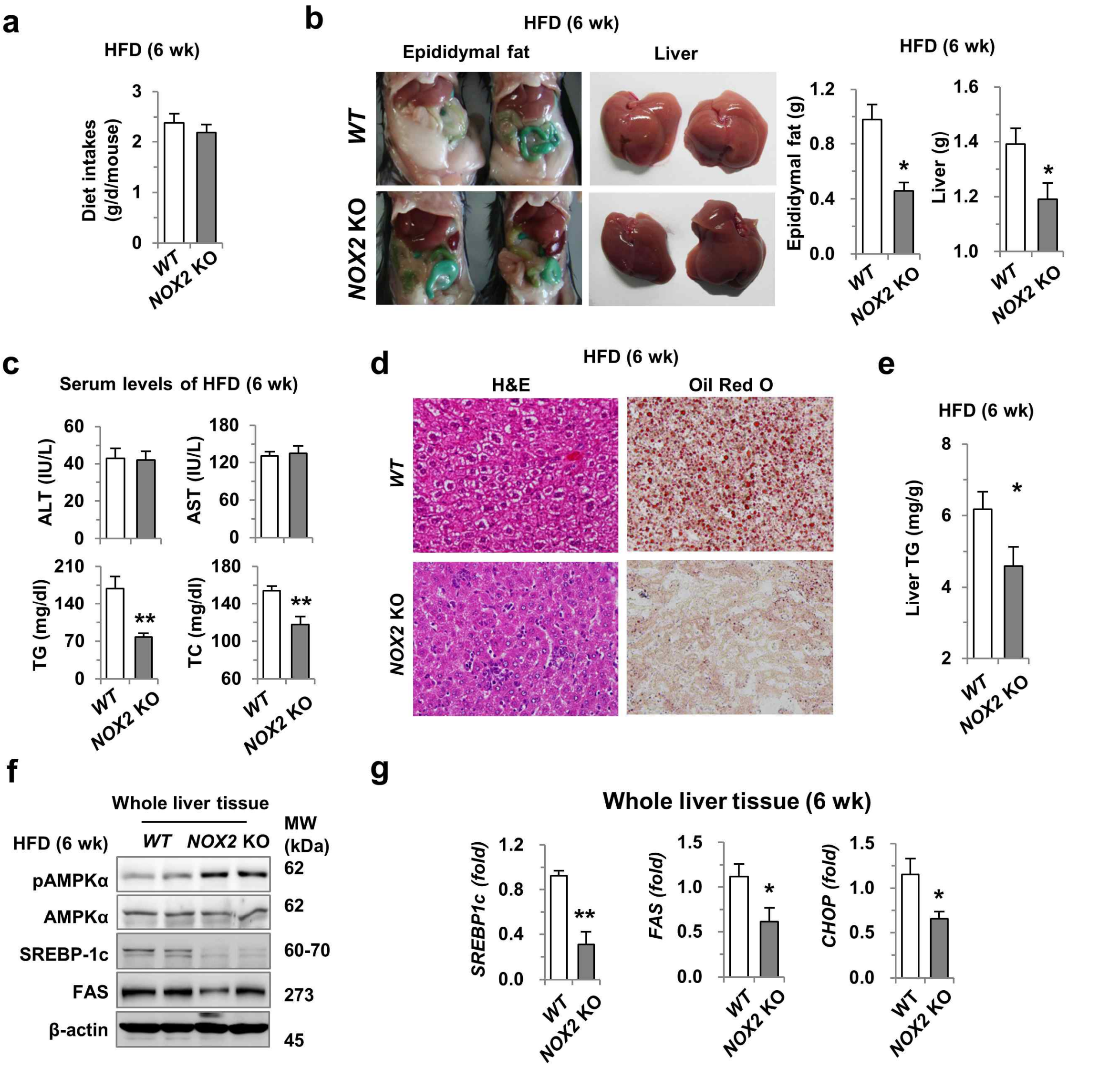

Supplementary Figure 1 │ Depletion of NOX2 ameliorates high-fat diet-induced hepatic steatosis in mice. Wild type (*WT*) and *Nox2* knockout (KO) mice are fed with high-fat diet for 6 weeks. (**a**) The amount of diet intakes. (**b**) The representative gross findings and their weights of epididymal fat and liver in *WT* and *Nox2* KO mice at week 6. (**c**) Blood chemistry analyses for ALT, AST, TG and TC. (**d**) Sectioned liver tissues are stained with H&E and Oil-Red O (×400). (**e**) TG levels are measured in whole liver tissues. (**f**) Liver tissues are subjected to Western blotting. (**g**) Real-time PCR analyses of whole liver tissues. Data are representative of three independent experiments using 5 mice per group. Data are expressed as the mean ± s.e.m. and analyzed by Student's *t*-test, $*P < 0.05$, $**P < 0.01$ in comparison with the corresponding controls.

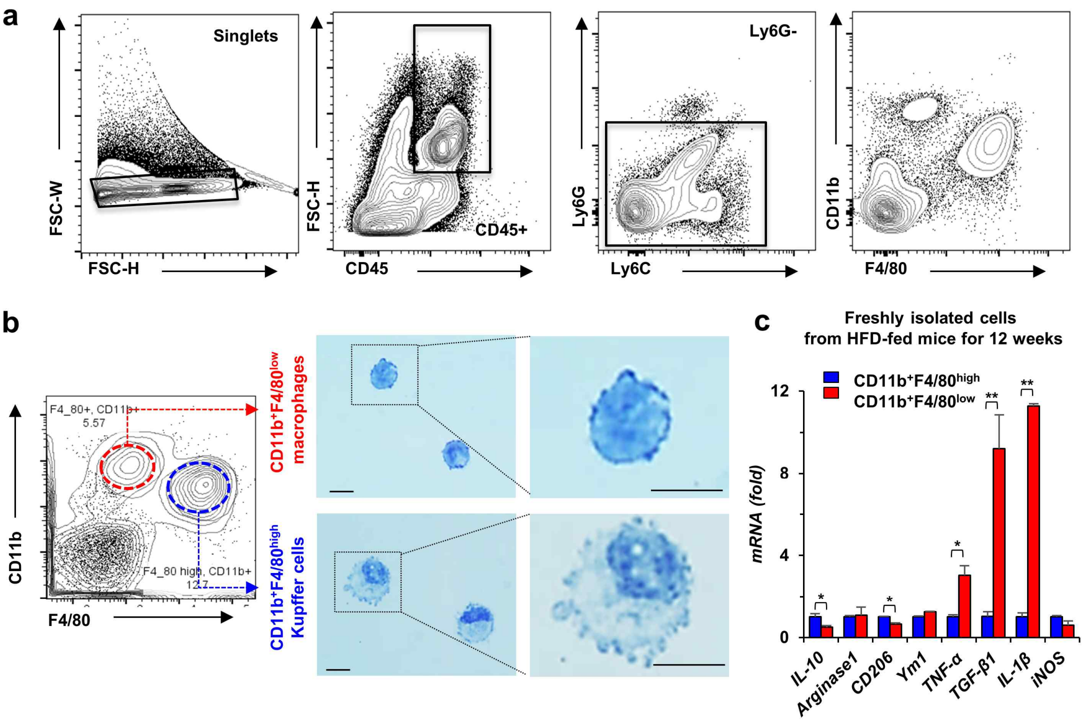

Supplementary Figure 2 | CD11b$^+$F4/80^{low} macrophages present more pro-inflammatory phenotype than CD11b$^+$F4/80^{high} Kupffer cells in mouse liver. (**a**) Flow cytometry analysis of freshly isolated liver mononuclear cells (MNCs). After gating singlet, CD45$^+$ cells were analyzed with antibodies of Ly6G and Ly6C. Then, CD45$^+$Ly6C$^+$Ly6G$^-$ cells are further analyzed with monocyte lineage markers F4/80 and CD11b. (b) CD11b$^+$F4/80^{high} Kupffer cells and CD11b$^+$F4/80^{low} macrophages were isolated individually. Isolated cells were visualized by Giemsa staining. Bar = 10 μm. (**c**) The gene expression of Kupffer cells and macrophages were assessed by qRT-PCR. (**c**) Relative expression of pro-inflammatory (M1) and alternative (M2) marker genes in Kupffer cells and macrophages isolated from mouse liver fed with high fat diet for 12 weeks. Data are representative of three independent experiments using isolated liver immune cells from 3 (**a,b**) and 5 (**c**) mice per group. Data are expressed as the mean ± s.e.m. and analyzed by Student's *t*-test, *P < 0.05, **P < 0.01 in comparison with the corresponding controls.

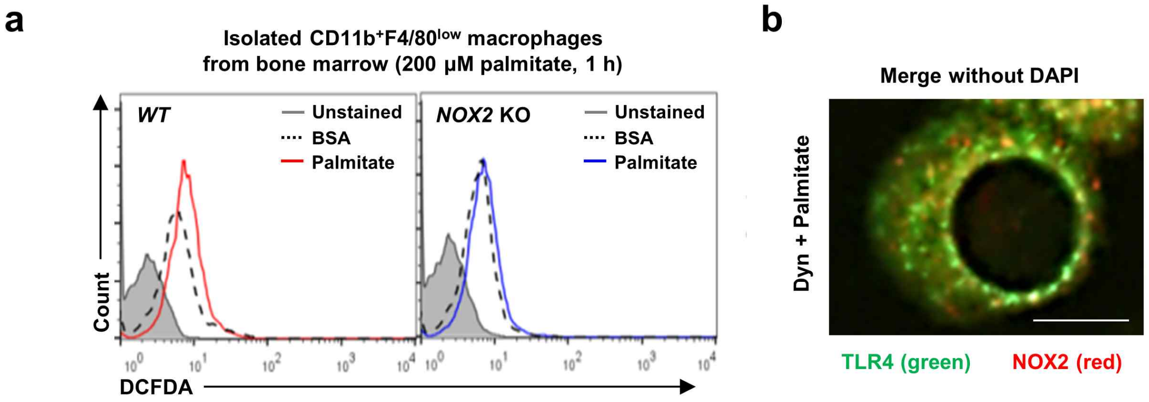

Supplementary Figure 3 │Palmitate treatment increases ROS generation in $CD11b^{+}F4/80^{low}$ macrophages by TLR4 and endocytosis-dependent manners. (**a**) Palmitate-mediated generation of reactive oxygen species (ROS) was monitored by DCF fluorescence in bone marrow-derived $CD11b^{+}F4/80^{low}$ macrophages of *WT* and *Nox2* KO mice. (**b**) RAW 264.7 macrophages were treated with palmitate (200 μM) ± dynasore (80 μM) for 1 hour. Then, these cells were subjected to immunostaining with antibodies of TLR4 and NOX2. Bar = 50 μm. Data are representative of three independent experiments *in vitro* using isolated liver immune cells from 3 (**a-c**) mice per group. Data are expressed as the mean ± s.e.m. and analyzed by Student's *t*-test, **P* < 0.05, ***P* < 0.01 in comparison with the corresponding controls.

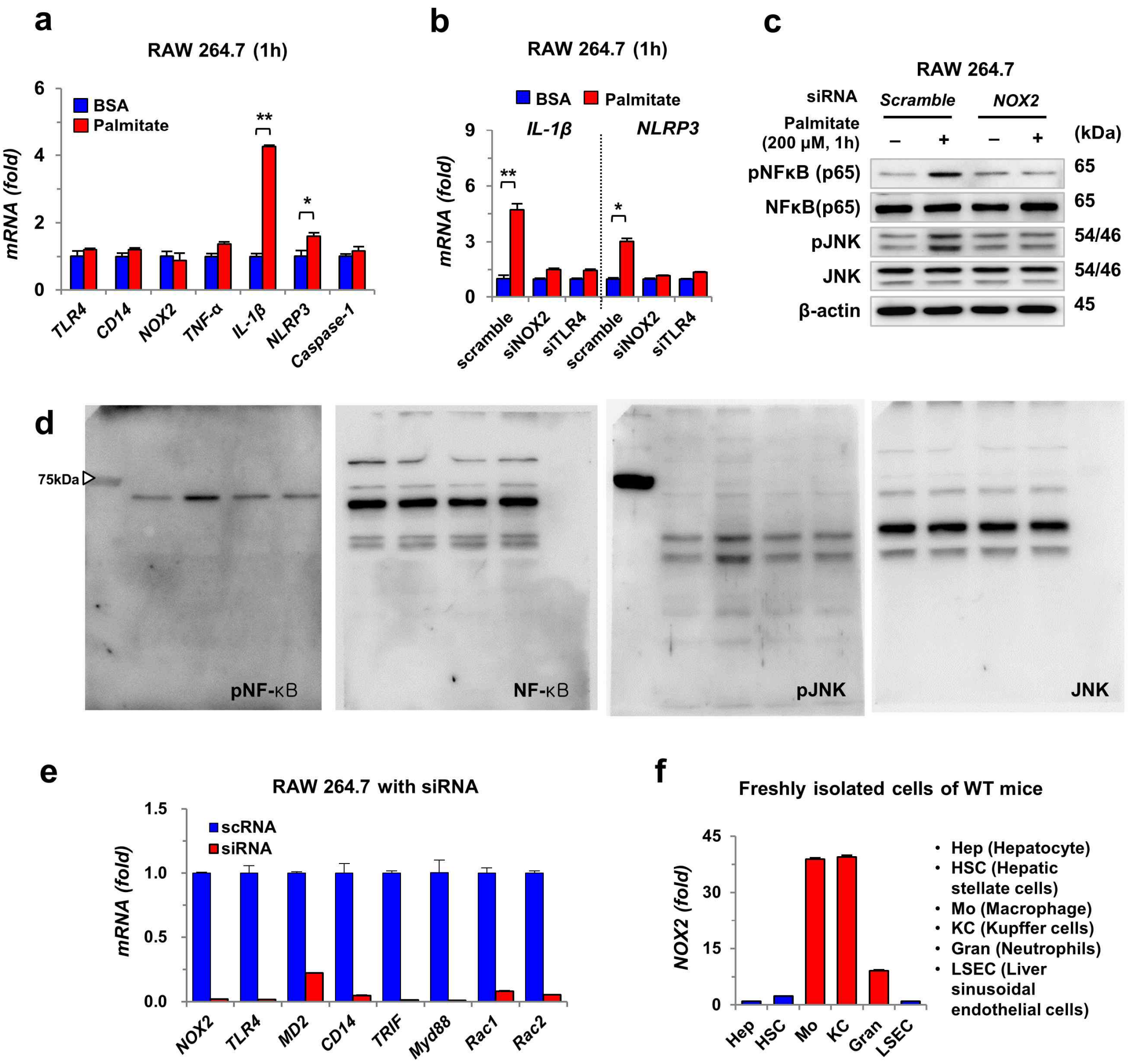

Supplementary Figure 4 │ Endocytosis of palmitate/TLR4-MD2 complex generates NOX2-mediated ROS by MyD88- and TRIF-independent manners. Raw 264.7 cells were stimulated with 200 μM palmitate for 1 hour. (**a**) RAW 264.7 cells treated with palmitate were subjected to qRT-PCR analyses. (**b-d**) After siRNA silencing and palmitate treatment, RAW 264.7 cells were subjected to qRT-PCR analyses (**b**) and Western blotting (**c,d**), respectively. (**e**) After siRNA silencing for *Nox2, Tlr4, Md2, Cd14, Trif, Myd88, Rac1* and *Rac2*, levels of mRNA were assessed. (**f**) *Nox2* expression was assessed in freshly isolated hepatic cells including hepatocytes, hepatic stellate cells, macrophages, Kupffer cells, neutrophils, and liver sinusoidal endothelial cells. Data are representative of three independent experiments. Data are expressed as the mean ± s.e.m. and analyzed by Student's *t*-test, $^*P < 0.05$, $^{**}P < 0.01$ in comparison with the corresponding controls.

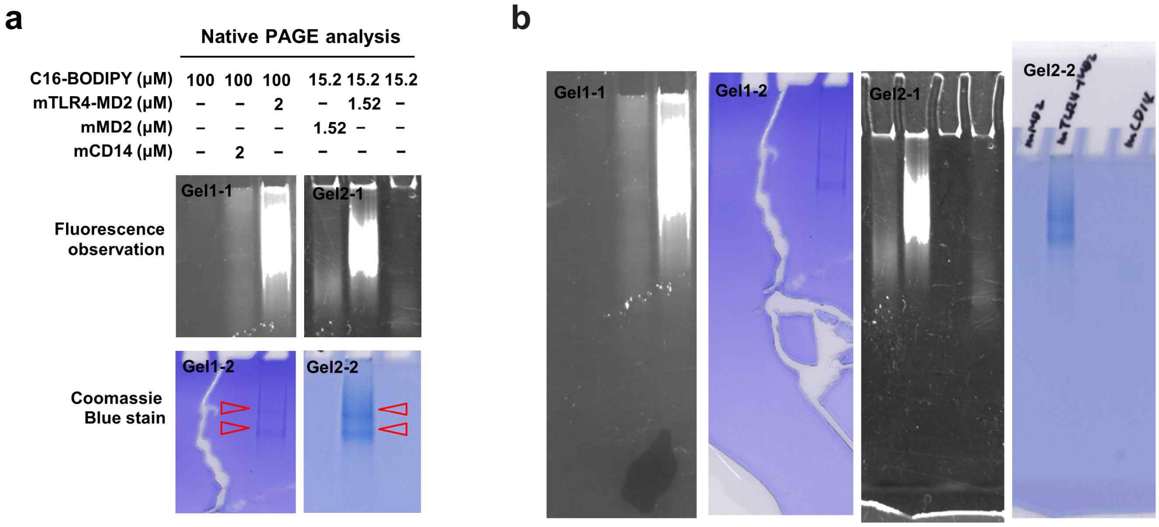

Supplementary Figure 5 │ Binding palmitate to the mouse TLR4-MD2 complex. After the reaction between each protein and BODIPY-labeled fluorescent fatty acid analogue (C16-BODIPY) under the indicated conditions, the samples loaded onto Native gradient PAGE (4-15%) were visualized with illumination at 488 nm and Coomassie staining, respectively. Data are representative of three independent experiments.

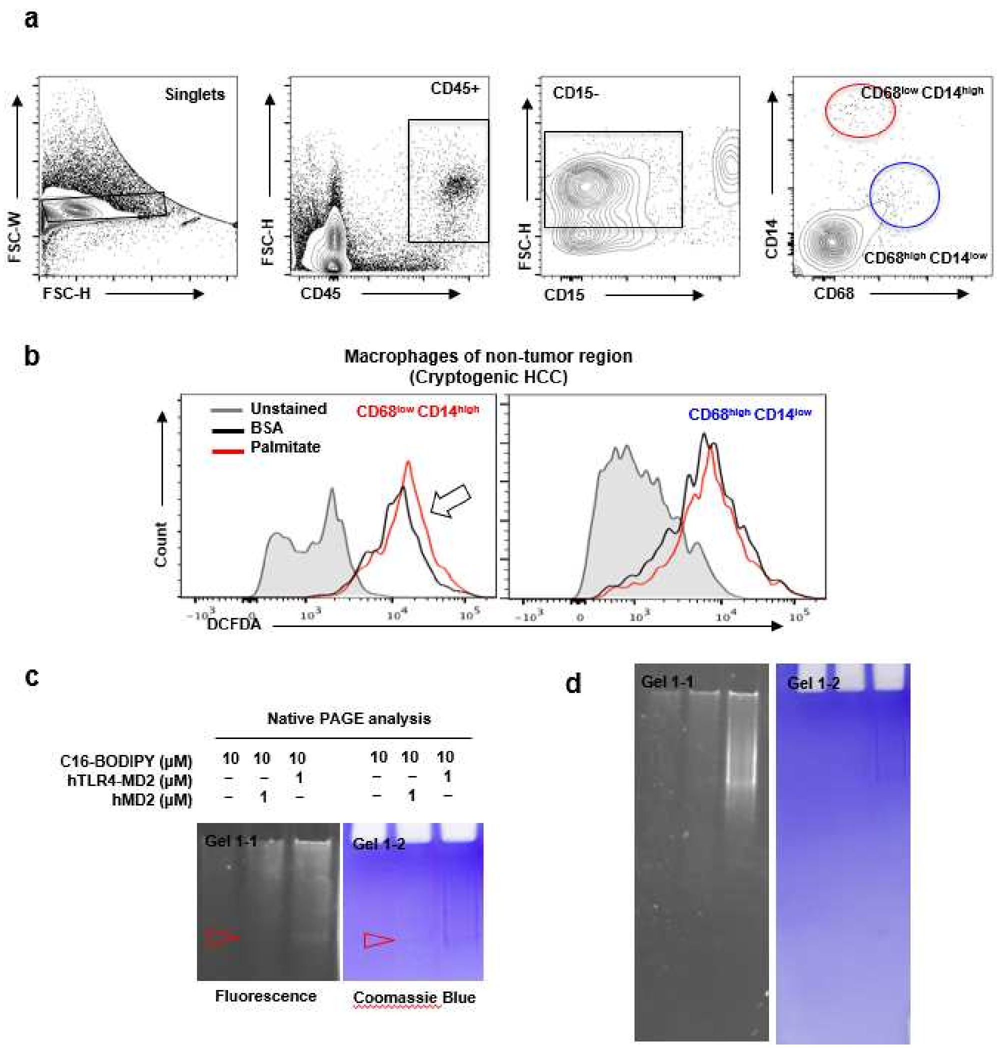

Supplementary Figure 6 │ Palmitate/TLR4-MD2 complex mediates NOX2-dependent ROS generation in non-resident hepatic macrophages in human. (**a**) Isolated human liver MNCs were analyzed by flow cytometry. After gating singlet, $CD45^+$ cells were analyzed with antibody of CD15. Then, $CD45^+CD15^-$ cells are further analyzed with monocyte lineage markers CD14 and CD68. (**b**) Isolated human liver MNCs from non-tumor lesions of primary hepatocellular carcinoma were treated with palmitate (200 μM) for 1 hour and then subjected to ROS measurement. Data are derived from liver MNCs of a cryptogenic HCC (n = 1) patient. (**c**) After the reaction between each protein and BODIPY-labeled fluorescent fatty acid analogue (C16-BODIPY) under the indicated conditions, the samples loaded at Native gradient PAGE (4-15%) were visualized with illumination (488 nm) and Coomassie staining.

GEO database (GSE63067)

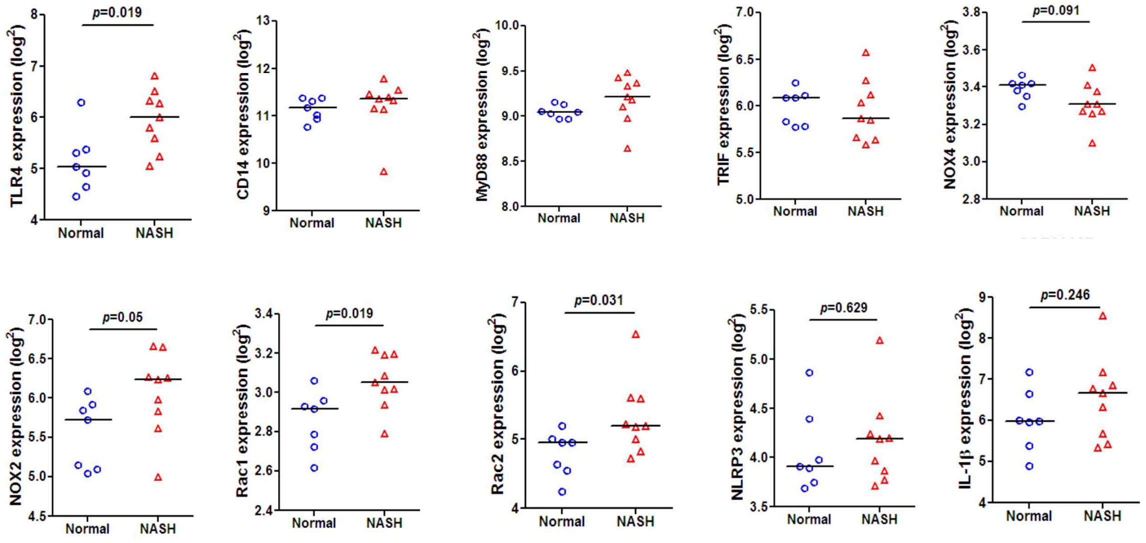

Supplementary Figure 7 │ Gene expression between healthy controls and NASH patients. Using GEO database (GSE63067), relative gene expression of T*lr4, Cd14, Myd88, Trif, Nox4,Nox2, Rac1, Rac2, Nlrp3, and Il-1 β* was compared between healthy controls and patients with NASH. Data are expressed as the mean ± s.e.m. and analyzed by Student's *t*-test.

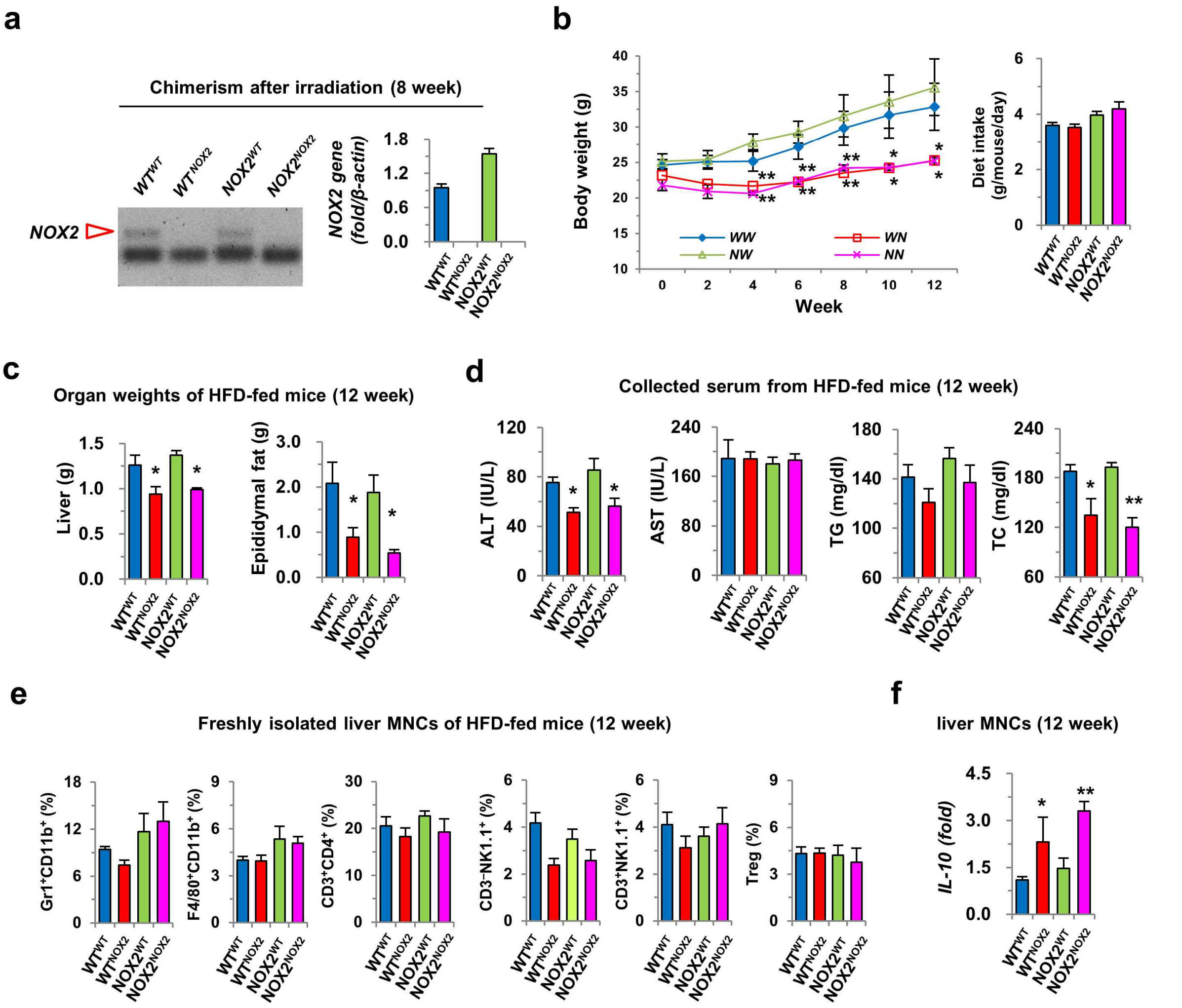

Supplementary Figure 8 │ NOX2-deficient bone marrow transplantation attenuates high-fat diet-induced hepatic steatosis in mice. After reciprocal bone marrow transplantation between *WT* and *Nox2* KO mice, mice were fed with high-fat diet for 12 weeks. (**a**) Chimerism of liver MNCs were assessed by RT- and qRT-PCR analyses. (**b**) Changes of body weights and diet intakes were measured. (**c**) Organ weights of liver and epididymal fat were measured. (**d**) Levels of ALT, AST, TG and TC were measured in collected sera. (**e,f**) Freshly isolated liver MNCs were subjected to flow cytometry and qRT-PCR analyses. Data are representative of two independent experiments using 5 mice per group. Data are expressed as the mean ± s.e.m. and analyzed by one-way analysis of variance, $*P < 0.05$, $**P < 0.01$ in comparison with the corresponding controls.

Supplementary Table 1. Oligonucleotide sequences of RNA used in siRNA transfection

Gene	Sense	Anti-sense
NOX2	GAUCUUAUGUGCAUAGAUA	UAUCUAUGCACAUAAGAUC
NOX4	UCAGACAAAUGUAGACACU	AGUGUCUACAUUUGUCUGA
TLR4	GAAUUGUAUCGCCUUCUUA	UAAGAAGGCGAUACAAUUC
Rac1	CAGUUCUAACUUAGCAAGU	ACUUGCUAAGUUAGAACUG
Rac2	ACUAGUGUGUUCGCCAUGU	ACAUGGCGAACACACUAGU
MyD88	CUCAACCCGUGUUCAAUGA	UCAUUGAACACGGGUUGAG
TRIF	GCAUUCUAGGUGCCUUGGA	UCCAAGGCACCUAGAAUGC
MD2	CUAUUCAUCAGUGUCAACU	AGUUGACACUGAUGAAUAG
CD14	CCUCGUCAACGAAUCCUCU	AGAGGAUUCGUUGACGAGG

Supplementary Table 2. Primer information

Gene	Forward (5'-3')	Reverse (5'-3')	Base pair
Mouse			
18S	ACAGGATTGACAGATTGATAGC	GCCAGAGTCTCGTTCGTTA	114bp
Arginase1	CTCCAAGCCAAAGTCCTTAGAG	AGGAGCTGTCATTAGGGACATC	185bp
AP-2	CCGACATCCGAAACTGTAAAAGT	GCTTCCATGTGTCCAAAGTCA	184bp
β-actin	GTTACCAACTGGGACGAC	CTCAAACATGATCTGGGTCA	150bp
Caspase-1	ACAAGGCACGGGACCTATG	TCCCAGTCAGTCCTGGAAATG	237bp
CB1R	ACAGGGCAGTACCCCTTCTT	AGCCCCTGGTGGTATTCTCT	175bp
CD14	CTCTGTCCTTAAAGCGGCTTAC	GTTGCGGAGGTTCAAGATGTT	191bp
CD206	CTCTGTTCAGCTATTGGACGC	CGGAATTTCTGGGATTCAGCTTC	132bp
CHOP	CTCCTGTCTGTCTCTCCGGAA	TACCCTCAGTCCCCTCCTCA	100bp
Clathrin	AGATTCTGCCCATTCGCTTTC	TCAGTGCAATCACTTTGCTGG	240bp
Dynamine2	AAGAGCCGAGTTTGAAGTGTG	ACGACTGCTCAATGTCAATCAG	192bp
FASN	TGGGTTCTAGCCAGCAGAGT	ACCACCAGAGACCGTTATGC	158bp
IL-1β	GCCCATCCTCTGTGACTCAT	AGGCCACAGGTATTTTGTCG	191bp
IL-6	TCCATCCAGTTGCCTTCTTG	TTCCACGATTTCCCAGAGAAC	166bp
IL-10	CGCAGCTCTAGGAGCATGTG	GCTCTTACTGACTGGCATGAG	105bp
iNOS	GTTCTCAGCCCAACAATACAAGA	GTGGACGGGTCGATGTCAC	127bp
NLRP3	ATTACCCGCCCGAGAAAGG	TCGCAGCAAAGATCCACACAG	141bp
NOX2	ACTCCTTGGGTCAGCACTGG	GTTCCTGTCCAGTTGTCTTCG	160bp
SREBP1c	CCTAACGTGGGCCTAGTCCGA AGCC	CCAGTTCGCACATCTCGGCCA	106bp
TLR4	AAATGCACTGAGCTTTAGTGGT	TGGCACTCATAATGATGGCAC	104bp
TGF-β1	TTGCTTCAGCTCCACAGAGA	TGGTTGTAGAGGGCAAGGAC	182bp
TNF-α	AAGCCTGTAGCCCACGTCGTA	AAGGTACAACCCATCGGCTGG	140bp
Ym1	CAGGTCTGGCAATTCTTCTGAA	GTCTTGCTCATGTGTGTAAGTGA	197bp
**Human**			
18S	ATCACCATTATGCAGAATCCACG	GACCTGGCTGTATTTTCCATCC	93bp
β-actin	AGCGAGCATCCCCCAAAGTT	GGGCACGAAGGCTCATCATT	285bp
CD14	ACTCCCTCAATCTGTCGTTCG	TCCCGTCCAGTGTCAGGTTAT	150bp
IL-1β	GGGACAGGATATGGAGCAACA	TTTCAACACGCAGGACAGGTA	127bp
NLRP3	CAGCCACCTCACTTCCAGTTT	CCCCAACCACAATCTCCGAAT	169bp
NOX2	ACACCCTTCGCATCCATTCTC	GCAAACCACTCAAAGGCATGT	125bp
Rac1	CGCAAACAGTTGGAGAAACGT	ATCGGCAATCGGCTTGTCTTT	68bp
Rac2	TCTCATCAGCTACACCACCAAC	GACGAGGGAGAAGCAGATGAG	199bp
TLR4	GGTCCTCAGTGTGCTTGTAGT	TTCCTTACCCAGTCCTCATCCT	159bp
TNF-α	TGGCGTGGAGCTGAGAGATAA	TTGATGGCAGAGAGGAGGTTGA	177bp
